# Ranolazine: a potential anti-metastatic drug targeting voltage-gated sodium channels

**DOI:** 10.1038/s41416-024-02622-w

**Published:** 2024-02-29

**Authors:** Mustafa B. A. Djamgoz

**Affiliations:** 1 Department of Life Sciences, Imperial College London, South Kensington Campus, London, SW7 2AZ UK; 2 Biotechnology Research Centre, Cyprus International University, Haspolat, Nicosia, TRNC, Mersin, 10 Türkiye

**Keywords:** Cancer, Oncology

## Abstract

**Background:**

Multi-faceted evidence from a range of cancers suggests strongly that de novo expression of voltage-gated sodium channels (VGSCs) plays a significant role in driving cancer cell invasiveness. Under hypoxic conditions, common to growing tumours, VGSCs develop a persistent current (I_NaP_) which can be blocked selectively by ranolazine.

**Methods:**

Several different carcinomas were examined. We used data from a range of experimental approaches relating to cellular invasiveness and metastasis. These were supplemented by survival data mined from cancer patients.

**Results:**

In vitro, ranolazine inhibited invasiveness of cancer cells especially under hypoxia. In vivo, ranolazine suppressed the metastatic abilities of breast and prostate cancers and melanoma. These data were supported by a major retrospective epidemiological study on breast, colon and prostate cancer patients. This showed that risk of dying from cancer was reduced by ca.60% among those taking ranolazine, even if this started 4 years after the diagnosis. Ranolazine was also shown to reduce the adverse effects of chemotherapy on heart and brain. Furthermore, its anti-cancer effectiveness could be boosted by co-administration with other drugs.

**Conclusions:**

Ranolazine, alone or in combination with appropriate therapies, could be reformulated as a safe anti-metastatic drug offering many potential advantages over current systemic treatment modalities.

## Background

Most cancers are carcinomas and, overall, the main cause of death from cancer is metastasis which necessitates systemic treatment. Although advances are constantly being made in the clinical management of metastatic disease, several limitations remain especially as regards early, functional diagnosis, and effective and durable therapies with minimum side effects. A relatively recent development in oncology is the discipline of ‘cancer neuroscience’ [1]. This involves (i) using neuroscience concepts and techniques to understand the intricacies of the cancer process drawing benefits from several decades of research on the nervous system and (ii) aiming to exploit cancer’s ‘neuronal’ properties to combat the disease. Cancer neuroscience falls into two broad areas: extrinsic and intrinsic. *Extrinsic* refers to the fact that many tumours are under the influence of the nervous system, either directly (through innervation) or indirectly (via circulating neurochemicals). This aspect has been reviewed extensively elsewhere [2] and is not considered further here. *Intrinsic* relates to the carcinomas’ inherent ‘neuronal’ characteristics [3]. The latter include the expression of molecular mechanisms like neuronal cell adhesion molecule, neurone-restrictive silencing factor and voltage-gated ion channels.

We have discovered that (i) cancer cells that are capable of metastasis have electrically excitable membranes and (ii) it is the electrical excitation that makes cancer cells aggressive, hyperactive, invasive and, ultimately, metastatic; we call this the ‘Celex Model’ (celex = cellular excitability) [4]. At the core of this novel phenomenon is de novo expression of voltage-gated sodium channels (VGSCs) accompanied by downregulation of outward currents due mainly to the activity of voltage-gated potassium channels. This combination enables cancer cells to generate tetrodotoxin (TTX)-sensitive, all-or-none action potentials (APs). The latter are normally associated with the so-called ‘excitable’ tissues of the body such as nerves and muscles. Ribeiro et al. revealed using micro-electrode array recordings that human breast cancer MDA-MB-231 cells were spontaneously active with APs blocked by TTX [5]. In vivo, also, McCallum et al. showed that APs could be recorded superficially from mouse 4T1 breast tumours [6]. Although some of these APs could be coming from the nerve input to the tumour, it was likely that intrinsic tumour activity was involved [6].

The primary role of VGSCs is to facilitate influx of sodium which cells use for a range of physiological and metabolic role [7, 8]. In line with functional VGSC expression, the level of sodium in several tumour types (detected, for example, by clinical ^23^Na-MRI) has been shown to be significantly higher than in corresponding normal tissues [9, 10]. At the cellular level also, the intracellular sodium concentration is higher than normal, and this is reduced by treating the cells with TTX implying, that VGSC activity contributes to this higher level [11].

VGSC activity promotes tissue invasion, at least in part, by acidifying the cancer cells’ surroundings through sodium-hydrogen exchange [12]. Proteolysis loosens the extracellular space for cells to escape into. Such invasive behaviour can lead to intravasation and, ultimately, metastasis. Although VGSCs comprise a multi-gene family with 9 functional isoforms, mainly 2 of these appear to be dominant in most cancers studied to date. The first is Na_v_1.5 which is expressed in breast, colon and ovarian cancers as well as in astrocytoma, neuroblastoma and melanoma. The other is Na_v_1.7, expressed in prostate, stomach and non-small cell lung cancers. Work on Na_v_1.5-expressing cancers (mainly breast and colon cancers) has shown that the neonatal splice variant (nNa_v_1.5) is dominant [13–16].

Under physiological (normoxic) conditions, VGSCs open and close over a few milliseconds with micromolar levels of sodium entering the cells [17]. However, under conditions when blood flow is impaired, and hypoxia develops, VGSCs do not inactivate so rapidly, remaining open for much longer than usual (for between several hundreds of milliseconds and seconds). This generates a persistent sodium current—I_NaP_ (sometimes referred to as the ‘late’ sodium current). Although only around one-thousandth of the amplitude of the primary transient component (I_NaT_), the prolonged I_NaP_ can facilitate entry of an excessive (millimolar) amount of sodium into the cells [18]. This property has been studied mostly in heart where Na_v_1.5 is common and hypoxia can develop from impaired blood flow when cardiac dysfunction such as arrhythmia occurs. Na_v_1.7 is also capable of generating a persistent current but, to date, this has been studied much less [19].

For effects on the cancer VGSC / nNa_v_1.5 to be clinically significant, it is necessary to block the pathophysiological I_NaP_ but not the physiological I_NaT_. This is due to the fact that I_NaT_ drives the generation of APs that enable nerve conduction and muscle function including heartbeat in the healthy body. Selective blockage of I_NaP_ is possible using the anti-angina drug, ranolazine -the central theme of this perspective.

## Ranolazine: essential facts

Ranolazine (brand name: ‘Ranexa’) is a piperazine derivative [RS-43285; N-(2,6-dimethylphenyl)-2-[4-(2-hydroxy-3-(2-methoxyphenoxy)propyl]piperazin-1-yl]acetamide)]. Piperazines are organic compounds that consist of a six-membered ring containing two nitrogen atoms at opposite positions in the ring. Ranolazine is used in clinical management of chronic angina pectoris. It is taken orally as tablets starting usually with 500 mg twice daily and this can be titrated to 1000 mg twice daily as tolerated. The half-life is 7 h, the peak level in plasma is reached within 2 to 5 h, and steady-state concentration is achieved within 3 days. Ranolazine is metabolised in the liver mainly by CYP3A4 and CYP2D6, so inhibitors of these enzymes would be contraindicated (see Section ‘Clinical potential and conclusion’).

Two main targets and modes of action have been associated with ranolazine. Initially, this drug was shown to inhibit metabolism of fatty acids (FAs), which would enhance glucose oxidation and thus reduce lactic acid production [20]. Since acidity is generally associated with *progression* of cancer, such action could potentially produce anti-cancer effects. More significantly, ranolazine binds to the ‘local anaesthetic site’ on the cytoplasmic side of VGSCs [21]. In the Vaughan-Williams classification, it is a type 1d blocker of VGSCs and, as such, it selectively inhibits I_NaP_, reducing the intracellular sodium concentration and, in turn, intracellular calcium via Na-Ca exchange. At clinical doses, equivalent to 2–8 µmol/L [22], ranolazine has little effect on I_NaT_ and as such it has proven effective against cardiac pathologies such as angina pectoris [23]. We were originally attracted to I_NaP_ in order to explain the fact that tumours contain millimolar more sodium than corresponding healthy tissues whilst I_NaT_ would permeate only micromolar sodium. In addition, as already noted, I_NaP_ is promoted by hypoxia and aggressive tumours are known to be hypoxic [24, 25]. In fact, the state of hypoxia and the invasiveness of cancer are positively correlated [26]. Hypoxia-driven generation of reactive oxygen species (ROS) may be one mechanism that promotes I_NaP_ [27]. Hypoxia may also have a transcriptional effect on VGSCs expression, but this is outside the scope of the current perspective [28]. It is now generally accepted that inhibition of I_NaP_ is responsible mainly for the cellular effects of ranolazine, at least under hypoxic conditions [29]. Further insights to VGSC / I_NaP_ vs. FA metabolism as primary target of ranolazine are presented in Section ‘Anti-cancer drugs’. Possible side effects of ranolazine and interaction with other drugs are mentioned in Section ‘Clinical potential and conclusion’.

## Evidence for the anti-metastatic role of ranolazine

Evidence for an anti-invasive/anti-metastatic role of ranolazine is derived from a range of approaches to several cancers, as follows.

### In vitro evidence

Driffort et al. showed initially for human breast cancer MDA-MB-231 cells that 50 μM ranolazine (i) suppressed the cells’ pro-invasive morphology and (ii) reduced the focal extracellular matrix degradative activity [30]. Consistent with these effects, ranolazine significantly inhibited cellular invasiveness through Matrigel matrix. Lee et al. and Qiu et al. confirmed these observations and revealed in addition that the anti-invasive effect could occur independently of proliferation [31, 32]. In addition, Qiu et al. showed that the anti-invasive effect of ranolazine was dose dependent and could be obtained at concentrations as low as 2.5 μM under hypoxia [32]. Guzel et al. demonstrated for human colorectal cancer cells that (i) hypoxia significantly enhanced Matrigel invasion and (ii) ranolazine at clinical doses inhibited invasiveness, again without affecting proliferative activity or cell viability [15]. In human and rat prostate cancers, the dominant VGSC subtype was shown to be Na_v_1.7 [33, 34]. Rizaner et al. showed for strongly metastatic rat prostate cancer Mat-LyLu cells that ranolazine (i) inhibited Matrigel invasion under both normoxic and hypoxic conditions and (ii) reduced the percentage of cells in the lung metastases expressing Na_v_1.7 [35].

### In vivo evidence

Consistent results have been obtained from a number of independent studies testing the effects of ranolazine on metastasis in vivo. Driffort et al. used the mouse tail vein model of metastatic breast cancer to show that dissemination of MDA-MB-231 cells to lung was significantly reduced by systemic treatment with ranolazine (50 mg/kg given for 5 days/week for 8 weeks) [30]. Importantly, the same study showed that silencing Na_v_1.5 in the cells *completely* eliminated the lung metastasis. Bugan et al. used the Dunning model of rat prostate cancer to show that 2.5–5 μM ranolazine administered by gavage reduced metastasis to lungs by up to 63% in double-blind experiments [36]. In contrast, under the same conditions, primary tumorigenesis, i.e., proliferative activity, was not affected. Compared with the control treatment, ranolazine also reduced the percentage of cells in the metastases expressing Na_v_1.7, the main VGSC subtype expressed in prostate cancer. Previous work using the same animal model showed that inhibiting VGSC activity specifically by using TTX injected directly into primary tumours (to avoid systemic toxicity) also suppressed lung metastasis and improved survival by some 20% [37]. Guth et al. showed in the TRAMPC1 genetic mouse model of prostate cancer that ranolazine (i) suppressed tumour growth and (ii) stimulated anti-cancer immunity measured by decreased tumour CD8 + T-cells Tim3 content, increased macrophages and decreased blood myeloid immunosuppressive monocytes [38]. Finally, Lasheras-Otero et al. showed in a mouse model of melanoma that ranolazine suppressed liver metastases [39]. This was suggested to be due to detached and circulating melanoma cells rewiring lipid metabolism by upregulating the expression genes involved in transport and beta-oxidation of FAs [39]. The possible involvement of VGSC activity / I_NaP_ was not questioned although previous work showed that (i) melanoma cells express functional, TTX-sensitive VGSCs [40] and (ii) melanoma tumours can be hypoxic, and hypoxia promotes melanoma progression [41].

### Evidence from humans

Several studies have examined whether VGSC blockers (local anaesthetics, anticonvulsants, antiarrhythmics etc) might also affect the cancer process and survival in human patients [42]. The picture turned out to be rather mixed for agents that block VGSCs somewhat indiscriminately [43]. In a more recent focussed study, Fairhurst et al. evaluated several classes of clinical blockers targeting various specific kinetic components of VGSCs [44]. A key question was whether the life expectancy of cancer patients taking ranolazine for non-cancer indications (e.g., angina pectoris) would differ from that of patients not using ranolazine. Data from breast, colon and prostate cancers over a 10-year period were pooled. Of these ca. 54,000 patients, a subgroup of 165 individuals were found to have taken ranolazine at various stages of their cancer. Prescriptions had started either before diagnosis and extended afterwards or started on average 4 years post-diagnosis. This group was compared with patients who did not take ranolazine. For the study group taking ranolazine (in the concentration range used for treating angina pectoris), the hazard ratio was ca. 0.41 (i.e., risk of dying from cancer was reduced by 59%) and this effect was a highly significant (*p* < 0.001). For those patients who started taking ranolazine after diagnosis, the hazard ratio was slightly higher (0.54) but the beneficial effect was still significant (*p* < 0.01).

In conclusion, in vitro, in vivo and epidemiological evidence from cancer patients are consistent in demonstrating that ranolazine can potentially suppress cancer cells’ invasive/metastatic activity and may help increase life expectancy.

## Additional benefits of ranolazine use

As noted in the Introduction, systemic treatment of cancer is often accompanied by undesirable side effects including on heart and brain. This is true for both chemotherapy and biological therapies. There is some experimental evidence to suggest that ranolazine can alleviate such side effects at least in animal models. Thus, Tocchetti et al. showed that ranolazine protected against experimental doxorubicin cardiotoxicity in mice [45]. Interestingly, similar protective results were obtained with the Na^+^/Ca^2+^ exchanger inhibitor KB-R7943. These results were consistent with I_NaP_ inhibition (i) reducing the Ca^2+^ overload induced by chemotherapy and (ii) restoring redox balance. Trastuzumab (Herceptin) is a biological agent (antibody) that blocks ErbB2 receptors and is used routinely against HER-2 positive breast cancer. Unfortunately, however, trastuzumab can cause heart muscle damage [https://www.cancerresearchuk.org/about-cancer/treatment/drugs/trastuzumab]. Riccio et al. demonstrated that in neonatal rat ventricular myocytes, trastuzumab significantly increased production of ROS [46]. Ranolazine could counteract this adverse effect and helped restore redox balance. In a brief, comparable study Chunchai et al. studied the effects of ranolazine on trastuzumab-induced ‘chemobrain’, characterised by overall cognitive decline, in Wistar rats [47]. Thus, treatment with trastuzumab caused brain mitochondrial dysfunction (ROS production, swelling) and dendritic contraction. These effects were largely reversed by co-treatment with a human-equivalent dose of ranolazine (305 mg/kg/day).

Thus, ranolazine could be doubly useful in cancer treatment, both directly in suppressing metastatic tendency and indirectly as a suppressor of some of the adverse side effects of chemotherapies and biological therapies used against cancer.

## Drug combination strategies

Attempts have been made to combine ranolazine with other drugs and thus reduce its effective dosage and possible side effects.

### Propranolol

This is a beta blocker which has been shown to have anti-cancer effects on various carcinomas [48]. Interestingly, it is also an effective blocker of neuronal and cardiac VGSCs [49]. So, on the surface, it would seem ideal to combine it with ranolazine. Propranolol by itself was highly effective in suppressing the invasiveness of the MDA-MB-231 human breast cancer cells, as shown earlier for other cancers [31, 48]. However, in combination with ranolazine, it proved antagonistic, i.e., ranolazine + propranolol co-treatment was significantly (ca. 30%) less effective than ranolazine alone [31]. One possible reason for this antagonism is that the two agents may compete for the same binding region on the VGSC protein, the cytoplasmic local anaesthetic binding site.

### Minoxidil

This is a potassium (K_ATP_) channel opener [50]. In a xenograft model of human ovarian cancer, minoxidil inhibited metastasis by promoting caspase-3 independent cell death [51]. Similarly, human breast cancer invasiveness in vitro was inhibited dose dependently by minoxidil [32]. The latter study also showed that ranolazine + minoxidil co-treatment was up to ca. 40% more effective than ranolazine alone [32]. In fact, in the presence of minoxidil, ranolazine proved effective at concentrations as low as 625 nM under hypoxic conditions. If these results could be extrapolating to the clinic, this would mean that in combination with minoxidil, the effective dose of ranolazine could be reduced by some 10-fold. The synergy with minoxidil is probably due to the fact that the two drugs have distinct targets, in overall agreement with the Celex model.

### Anti-cancer drugs

Interestingly, in a recent different approach, ranolazine was combined with immunotherapy. Thus, Redondo-Muñoz et al. used an in vivo mouse model of melanoma to show that combining PD-L1 antibody treatment with ranolazine led to significantly improved survival [52]. This effect involved enhanced anti-tumour immune responses (increased antigen presentation and interferon signalling) via FA metabolism but not a VGSC. The reason(s) for the apparent lack of involvement of a VGSC in this study is not clear at present. It is possible that both FA oxidation and VGSC / I_NaP_ inhibition are targeted by ranolazine, depending on the type of cancer (and possibly species) and, importantly, the stage of the cancer, for example with regard to hypoxia. Thus, in the experiments of Guzel et al. [15] on human colorectal cancer cells ranolazine inhibited invasion under hypoxia and silencing the VGSC (nNa_v_1.5) eliminated the effect of ranolazine. In contrast, in the study of Redondo-Muñoz et al. [52] on human and mouse melanoma cells and mouse xenografts, the anti-cancer effect of ranolazine was shown to be independent of any VGSC. More work is needed to generalise these issues.

Riluzole is another I_NaP_ blocker [53] shown to have anti-invasive effects [35]. It could also potentiate the effectiveness of anti-cancer drugs. Thus, in combination with either fulvestrant or 4-hydroxytamoxifen, riluzole additively suppressed oestrogen receptor positive breast cancer cell growth in vitro. In ex vivo primary breast tumour explant cultures, riluzole significantly enhanced the anti-proliferative effect of fulvestrant [54]. Cisplatin resistance in colorectal cancer cells was shown earlier to be overcome, at least partially, by treatment with riluzole [55].

In conclusion, ranolazine (and riluzole) could also be used to potentiate the effectiveness of other anti-cancer drugs but the combinations to be tested must be mechanistically meaningful in order to avoid possible interference.

## Clinical potential and conclusion

From the available evidence, taken together, we can propose that ranolazine could be utilised as an anti-metastatic drug [56]. Indeed, the evidence, at all levels from in vitro to human, is consistent for the role of ranolazine in suppressing cellular invasiveness and full-blown metastasis in several different carcinomas. Ranolazine has been in clinical use against angina pectoris for several years and, more recently, it has proven to be highly effective also against arrhythmia. Thus, it has a well-known dosage and safety profile. Nevertheless, some adverse side effects have been reported, including dizziness, headaches, nausea, debility and constipation [https://www.ncbi.nlm.nih.gov/books/NBK507828/]. Furthermore, it is recommended that ranolazine is not used together with some drugs such as other VGSC modulators (e.g., carbamazepine, phenytoin), as mentioned above for propranolol [31], antidepressants (e.g., nefazodone, amitriptyline), anticonvulsants (e.g., phenobarbital), CYP3A4 inhibitors (e.g., ketoconazole, diltiazem, verapamil), and anti-fungals/bacterials (e.g, itraconazole, ketoconazole, clarithromycin) [https://www.ncbi.nlm.nih.gov/books/NBK507828/]. There are also some reports of ranolazine inducing long QT, but this is likely to occur only at higher doses [57].

Another advantage of targeting I_NaP_ is the fact that the VGSC generating this current is essentially a *functional* biomarker. Accordingly, patients can readily be stratified by their profile of VGSC protein expression by immunohistochemical staining of their biopsies which is done routinely in hospital pathology laboratories. Ranolazine therapy would be appropriate only for patients whose biopsies were found to express VGSC protein. In this regard, we have already developed a polyclonal antibody specific for nNa_v_1.5 [58] and a novel monoclonal antibody is currently being validated.

In overall conclusion, ranolazine has the potential to be readily adopted as an anti-metastatic drug, either by itself or in appropriate combination with a mechanistically compatible agent and this can be done as *precision medicine*. Further studies are justified to examine whether ranolazine could keep tumours in a localised state and thus enable patients to live with their cancer as a chronic disease, as with chronic angina.
